# Using the Findings of a National Survey to Inform the Work of England’s Genomics Education Programme

**DOI:** 10.3389/fgene.2019.01265

**Published:** 2019-12-17

**Authors:** Siobhan Simpson, Anneke Seller, Michelle Bishop

**Affiliations:** Genomics Education Programme, Health Education England, Birmingham, United Kingdom

**Keywords:** training needs assessment, genomics education, genomic medicine, survey, education strategy, multidisciplinary

## Abstract

A national coordinated approach to workforce education and training in genomics is essential for the successful implementation of whole genome sequencing and, more broadly, genomic medicine within the National Health Service (NHS) in England. However, there have been no workforce wide assessments of genomics education and training needs that can be used to inform the strategic approach to be taken. In order to assess these needs the Genomics Education Programme (GEP) undertook a cross-professional training needs analysis. Responses from 2,814 individuals allowed the identification of four themes related to NHS staff's perceived education and training needs in genomics, those who: a) have a role in genomics and are competent; b) have a role in genomics but identified a specific learning need; c) could not identify whether genomics is relevant, but want to know more, and; d) do not see genomics as relevant to their role and do not believe they need to learn about it. Individuals are motivated to undertake training for their own continuing professional development and if they perceive training to have a direct impact on patient care. Overall, online learning is the preferred mode of delivery, but there are still many individuals who value face-to-face teaching. This paper demonstrates how the GEP has used these findings to provide an evidence base to inform the ongoing strategy for genomics education and training in the NHS, including the development of competency frameworks and a range of resources to address the diverse genomics learning needs of the healthcare workforce.

## Introduction

Genomics has been a focus within England's National Health Service (NHS) since the launch of the landmark 100,000 Genomes Project in 2012 (Couzin-Frankel, 2012). The information learned from this project is now informing the development and implementation of an England-wide NHS Genomic Medicine Service (NHS England, 2019b). This service will increase access to genomic testing across different specialties so clinicians can use this technology as part of the patient diagnosis, treatment, and management pathway.

Organizations responsible for the training and regulation of healthcare professionals in the UK recognize the impact of genomics in healthcare, and therefore the importance of genomics education and training for future healthcare staff. This is evidenced by the embedding of genomics into relevant professional standards and training programs (General Medical Council, 2018; Nursing and Midwifery Council, 2018; National School of Healthcare Science, 2019). However, with genomic testing now entering mainstream care (NHS England, 2019b), an understanding of the technology and the information these tests provide is needed by many of the NHS's current 1.4 million staff. The level of understanding required will differ depending on the role undertaken by the individual. This may range from an awareness of genomics and how genomics is used in their area of practice, through to specialist knowledge on testing, interpretation of results, and how genomics influences patient care and management. This poses a challenge: to provide appropriate education and training for the existing NHS workforce, across multiple professional groups in a rapidly changing field.

Health Education England (HEE) is responsible for improving the quality of patient care in the NHS through education, training, and development of NHS staff in England (www.hee.nhs.uk). The Genomics Education Programme (GEP), which sits within HEE, is the NHS in England's method of ensuring its staff have the knowledge, skills, and experience to ensure that the health service remains a world leader in genomic and precision medicine (www.genomicseducation.hee.nhs.uk).

To effectively provide education and training for the workforce, an understanding of the areas in which the workforce requires development is needed. Previous studies of genetics and genomics training needs of the healthcare workforce have identified gaps in knowledge and training but have tended to focus on a single workforce group rather than a whole healthcare system. Workforce groups such as physicians and nurses have been assessed, but often in specific areas—for example, with regards to direct to consumer testing, pharmacogenetics, or whole genome sequencing (Powell et al., 2012; Selkirk et al., 2013; Christensen et al., 2016), or particular specialisms—for example, obstetrics and gynecology, or dermatopathology (Adjei et al., 2017; Torre et al., 2018). There is acknowledgement, however, from those within other healthcare professions of a need for genomics education, but little direct evaluation of their training needs has been undertaken (Cornwall et al., 2018). Most studies have been conducted outside the UK or if done within the UK they have focused on a specific workforce group (Godino and Skirton, 2012).

While it is likely that these results are applicable across the NHS workforce it cannot be assumed they reflect the wider situation within England. As a first step in establishing a strategic approach to ensure all NHS staff can access genomic education and training that meets their learning and professional needs, the GEP undertook a cross-professional training needs analysis to identify genomic learning requirements across this large and diverse group.

## Methods

### NHS Workforce and Genomic Medicine Centres

#### NHS Workforce

The NHS workforce is large and diverse, with 1,390,849 people employed by NHS England (NHS Digital, 2018). Of these 11.5% are doctors (n=160,135), 23.0% have a nursing qualification (n = 320,324), 1.9% are midwives (n = 25,866), and 1.6% are ambulance staff (n = 22,245). In addition, 11.5% are classified as scientific, therapeutic, and technical staff (n = 159,674). The remaining 50.5% of NHS staff have roles supporting clinical staff and in auxiliary services such as the operational and infrastructure side of the NHS (NHS Digital, 2018).

#### Genomic Medicine Centres

Thirteen Genomic Medicine Centres (GMCs) were established by NHS England between 2014 and 2015 to support the delivery of the 100,000 Genomes Project. These Centres covered all geographical areas of England (see **Supplementary Material**) to ensure equitable access to the project for eligible NHS patients (Genomics England, 2018). Within each of the GMCs, an Education and Training Lead was appointed to facilitate local workforce development in genomics, both within and outside of the GMC. The GEP provided financial support and oversight of the education and training activities within each GMC.

### Data Collection

To inform regional and national strategies for NHS workforce development in genomics, the Education and Training Lead in each GMC was tasked to develop a questionnaire to identify local requirements. The GEP was informed that NHS ethics approval was not required as the purpose of these surveys was service evaluation. Handling of data was carried out within the governance framework of each organization. General guidance on the purpose and structure of the questionnaire was provided by the GEP, but the GEP did not directly design or deploy any questionnaires. Thus, each of the GMC regions developed their own questionnaire enabling different service requirements to be addressed within the surveys. Questionnaires were entered into either Survey Monkey or Bristol Online Survey system. Electronic links to the surveys were deployed through different communication networks available to the Education and Training leads within their regions, such as hospital trust intranets and mailing lists. Where possible, reminders were sent. Due to the different methods in which the surveys were deployed, it is not possible to determine the number of NHS staff who received the link to the online surveys. Data collection occurred between July 2016 and April 2017. Two questionnaires targeted specific workforce groups (West Midlands and Yorkshire and Humber) as these were considered workforce development priority areas for these regions, while the other questionnaires were aimed at the NHS workforce more generally. An exemplar questionnaire is available from the authors on request.

### Measures

Each questionnaire had between 9 and 20 questions. Here we present the findings related to questions asking about perceived education and training needs, training delivery preferences, and motivations to undertake training.

Demographics, including involvement in genomics, were collected using closed questions [for example: “are you involved in the 100,000 Genomes Project” (Yes/No/Don't know), “apart from the 100,000 Genomes Project, do you currently have a role in delivering any genetics/genomic services (Yes/No)”]. Two questionnaires asked about use of genomics in current practice by asking a series of statements: “are you currently using genomics in your clinical practice for prevention/diagnosis/treatment/No/Not applicable for my role.” Another questionnaire asked more specific questions around involvement with “genetic testing” (Yes/No), “discussion of genomics or molecular diagnostics at MDT” (Yes/No), and “processing samples for 100,000 Genomes Project” (Yes/No). Previous training in genetics and genomics was asked by four of the questionnaires by asking “Have you had any previous training in genetics and/or genomics?” followed by a list where respondents could tick as many as applied.

*Education and training needs:* Perceived knowledge and skill gaps were asked in three different ways: “Do you feel you have sufficient knowledge and the skills to perform your current role in genetics/genomics?” (Yes/No/My role does not involve genetics/genomics); “Do you feel you have sufficient knowledge in genomics to allow you to do your job effectively?” (Yes/No); “Do you feel that you need further training in genomics?” (Yes/No).

*Training delivery preferences*: Five of the questionnaires asked, “How would you like training to be delivered?” followed by a list of options, with respondents able to tick as many as applied. Three questionnaires asked follow-on questions to the primary question about perceived education and training needs, to ask respondents to specify how they would like education and training to be delivered with a list of options provided.

*Training motivation*: Four questionnaires asked respondents “what motivates you to undertake education and training” with a list of options. Another questionnaire asked the same question but left this as a free-text response.

All questionnaires provided the option for free-text responses throughout to clarify or comment on their responses. In addition, four questionnaires also provided the opportunity for respondents to provide any closing remarks before exiting the questionnaire.

### Data Analysis

Data from the questionnaires were downloaded, anonymized, and sent to the GEP in an Excel format. Quantitative data from each questionnaire were analyzed separately. Descriptive statistics were used to describe the sample in terms of their professional workforce group, previous genomics education, and their perceived education and training needs in genomics. The responses to the question asking about education and training delivery methods were coded as “face-to-face,” “online,” or “both.” For statistical analysis only individuals who expressed a preference for one or the other (as opposed to "both") were analyzed. For four of the five questionnaires that asked about training motivations, descriptive statistics were used to describe the sample. For the fifth questionnaire, free text responses were coded to the categories used in the other questionnaires. Where possible, Kruskal-Wallis tests, with appropriate *post hoc* testing, were performed to determine an association between professional workforce groups and education and training needs, preferred education and training delivery methods, as well as motivation to participate in education and training. Thematic analysis of the free-text comments made throughout the questionnaires was conducted using a constant comparison approach as first described by Glaser and Strauss (1967).

## Results

A total of 2,814 responses were received from eight questionnaires (covering nine GMCs), representing 10 workforce groups (see **Table 1** for a description of the workforce groups). These workforce groups included clinical and non-clinical roles, as well as “other” individuals such as hospital chaplains, housekeepers, and librarians. Most responses were received from medical professionals (34.4%), with the least (less than 1%) from the public health workforce. Overall 880 (31.3%) respondents indicated they were currently involved in the delivery of genetic and/or genomic services, including the 100,000 Genomes Project (**Table 2**). Of those respondents asked about their previous education and training in genomics (n = 1625), 322 (19.8%) had no previous genomics education and training, 674 (41.5%) had undertaken CPD, 474 (29.2%) had genomics education as part of a non-specialized degree (e.g. undergraduate medical degree), and 155 (9.5%) had obtained a specialized genomics degree.

**Table 1:** Definitions of workforce groups.

Workforce group	Definition
Medical professionals	All levels and specialty of medical doctors, plus physician assistants.
Nurses, midwives, and associated roles	Nurses, midwives, nursing associates, and healthcare assistants.
Healthcare scientists	Any health professional who is registered as a clinical scientist, bioinformatician, genetic counsellor, biomedical scientist, or works in affiliated role such as a genetic technologist (as defined by Health Careers (2019).
Allied health professionals	Includes dietitian, speech and language therapist, physiotherapist, podiatrist, etc. For a full list of NHS allied health professionals see Health Careers (2019).
Administration and clerical	Administrators and secretaries.
Pharmacy professionals	Pharmacists, pharmacy technicians, pharmacy assistants, and medicines management technicians.
Healthcare managers	Managers of all types.
Researchers	Individuals with a direct research role.
Dentistry	Dentists and dental surgeons.
Public health worker	Self-defined by respondents.

**Table 2:** Total respondents in each workforce group from each questionnaire and how many of those respondents indicated that their role involves genomics (Genomics).

	East of England GMC		Oxford GMC		South West and West of England GMCs		West Midlands GMC		South London GMC		Greater Manchester GMC		Yorkshire and Humber GMC		North West Coast GMC		Total	
	Total	Genomics	Total	Genomics	Total	Genomics	Total	Genomics	Total	Genomics	Total	Genomics	Total	Genomics	Total	Genomics	Total	Genomics
Medical professionals	324	94 (29.0%)	53	25 (47.2%)	30	18 (60.0%)	0	0 (0%)	137	67 (48.9%)	262	113 (43.1%)	66	47 (71.2%)	97	50 (51.5%)	969	414 (42.7%)
Nurses, midwives, and associated roles	271	54 (19.9%)	89	19 (21.3%)	48	15 (31.3%)	1	1 (100%)	52	17 (32.7%)	93	6 (6.5%)	70	12 (17.1%)	70	24 (34.3%)	694	148 (21.3%)
Healthcare scientists	112	73 (65.2%)	71	34 (47.9%)	18	11 (61.1%)	194	73 (37.6%)	40	20 (50.0%)	63	19 (30.3%)	1	0 (0%)	22	12 (54.5%)	521	242 (46.4%)
Allied health professionals	68	5 (7.4%)	19	1 (5.3%)	8	1 (12.5%)	0	0 (0%)	12	3 (25.0%)	29	0 (0%)	0	0 (0%)	9	0 (0%)	145	10 (6.9%)
Administration and clerical	64	5 (7.8%)	11	1 (9.1%)	9	6 (66.7%)	0	0 (0%)	12	2 (16.7%)	24	0 (0%)	0	0 (0%)	12	1 (8.3%)	132	15 (11.4%)
Others	73	6 (8.2%)	8	0 (0%)	4	0 (0%)	0	0 (0%)	3	0 (0%)	5	1 (20.0%)	1	0 (0%)	2	0 (0%)	96	7 (7.3%)
Pharmacy professionals	51	3 (5.9%)	3	0 (0%)	6	1 (16.7%)	0	0 (0%)	7	1 (14.3%)	17	0 (0%)	0	0 (0%)	13	2 (15.4%)	97	7 (7.2%)
Healthcare managers	35	8 (22.9%)	6	1 (16.7%)	7	4 (57.1%)	2	1 (50.0%)	8	1 (12.5%)	16	1 (6.3%)	0	0 (0%)	10	2 (20.0%)	84	18 (21.4%)
Researchers	46	13 (28.3%)	5	3 (60.0%)	3	1 (33.3%)	0	0 (0%)	0	0 (0%)	7	2 (28.6%)	0	0 (0%)	0	0 (0%)	61	19 (31.1%)
Dentistry	4	0 (0%)	1	0 (0%)	0	0 (0%)	0	0 (0%)	2	0 (0%)	3	0 (0%)	0	0 (0%)	1	0 (0%)	11	0 (0%)
Public health worker	2	0 (0%)	1	0 (0%)	1	0 (0%)	0	0 (0%)	0	0 (0%)	0	0 (0%)	0	0 (0%)	0	0 (0%)	4	0 (0%)
Total	1050	261 (24.9%)	267	84 (31.5%)	134	57 (42.5%)	197	75 (38.1%)	273	111 (40.7%)	519	142 (27.4%)	138	59 (42.8%)	236	91 (38.6%)	2814	880 (31.3%)

### Identifying Learning Needs

Not all respondents who competed the questionnaires stated that they needed genomics education and training. **Table 3** outlines the results for each questionnaire. For the questionnaires that asked if respondents had sufficient knowledge in order to perform their role, between 5.1% and 40.8% replied no, indicating they needed further training. Conversely in those questionnaires that asked if they felt they needed further training in genomics, between 75.9% and 85.7% responded yes, they did need further training.

**Table 3:** Perceived education and training needs of NHS Healthcare Professionals.

Region	Yes (%)	No (%)	Total
“Do you feel you have sufficient knowledge and skills to perform your current role in genetics/genomics?”			
East of England GMC	162 (67.2%)	79 (32.8%)	241
South West and West of England GMCs	29 (61.7%)	18 (38.3%	47
“Do you feel you have sufficient knowledge in genomics to allow you to do your job effectively?			
Oxford GMC	151 (59.2%)	104 (40.8%)	255
West Midlands GMC	167 (84.8%)	30 (15.2%)	197
Yorkshire and Humber GMC	130 (94.9%)	7 (5.1%)	137
“Do you feel you need further training in genomics?”			
South London GMC	214 (81.4%)	54 (20.6%)	263
Greater Manchester GMC	445 (85.7%)	74 (14.3%)	519
North West Coast GMC	176 (75.9%)	56 (24.1%)	232

There were no significant differences in perceived need for further training between the workforce groups within each questionnaire with two exceptions: Oxford (Kruskal-Wallis p < 0.01) and Greater Manchester (Kruskal-Wallis p < 0.001). For the respondents from Oxford the significant test result is due to the difference between the nurses, midwives, and associated roles group (41.2% state sufficient knowledge) and the Healthcare scientists group (78.6% state sufficient knowledge) (Dunn's pairwise tests p < 0.001, adjusted using the Bonferroni correction). In the respondents from Greater Manchester the difference (Dunn's pairwise tests p < 0.01, adjusted using the Bonferroni correction) is between the Administration and clerical group and all other groups. Only 54.2% of the Administration and clerical group indicated that they would like more training, while the other groups were all over 82.8%. Neither involvement in delivering genetic/genomic services or the level of previous education and training were significantly associated with reported education and training need across the questionnaires.

Analysis of the free-text comments in each of the questionnaires identified four themes relating to NHS staff's education and training needs.

*A. Individuals have a role in genomics and are competent*. These individuals felt they had enough knowledge and the right skills to perform their current role; however, respondents were cognizant that genomic knowledge constantly evolves, and, as stated by one respondent:

*“There's always so much to learn”* (Nurse, Pediatrics).

There was also the recognition from some of these respondents that they were making a self-assessment of their competence and, as such, may not have all the knowledge and skills they need. As one medical professional commented:

*“But I might be unconsciously incompetent”* (Medical consultant, Immunology).

*B. Individuals have a role in genomics and identified a specific learning need*. While many of the learning needs quoted by respondents related to very niche areas of knowledge and specific skills, three common areas were identified:

- Core bioinformatic knowledge and skills
- Knowledge to support variant interpretation
- Genetic counselling skills

*C. Individuals could not identify whether genomics is relevant to their practice but want to know how genomics may impact on their clinical role*. Some of these respondents were aware that genomics would be relevant to their professional group, whereas others were not sure. However, both groups still wanted to find out more about the application of genomics to healthcare. In general, these respondents requested introductory level resources, primarily related to their professional group such as “genomics for nurses” and the “application with respect to radiology.”

*D. Individuals do not see genomics as relevant to their role and do not believe there is a need to learn about it*. These NHS staff were not interested in knowing more about genomics, as they could not see how it would change their every-day practice.

*“Do I need to know more? I can do my job without having any knowledge in genomics“* (Nurse, Intensive care)

However, it is likely that some of these responders will need some level of genomics knowledge, as genomics is being used in the clinical area in which they work (e.g., maternity, cardiology, pediatrics, etc.).

As the free-text questions were optional, counting the responses would not have provided a reliable indication of the proportion of healthcare professionals within each category.

### Challenges to Identifying a Learning Need

Analysis of the questionnaire comments also highlighted elements that made identifying genomic learning needs challenging. For some respondents, their lack of knowledge about genomics itself meant that they did not know if this was a topic they should know more about.

*“I honestly don't know, I have no idea what it is“* (Nurse, Anesthetics)

*“Not familiar with the term Genomics”* (Medical Consultant, Gynecology)

Others were quite skeptical on the impact of genomics, so questioned the relevance and the need for education and training in this area.

*“If the outcome is to tell patients to do anything other than lose weight, exercise and stop smoking and drinking, I will be astonished“* (General Practitioner)

For others, in particular those who responded to surveys where the question about education and training was directly linked to their current practice, a lack of clarity about their role made answering this question difficult.

### Training Delivery Approaches

All surveys (n = 2,814 respondents) asked a question around preferred method of learning. There were respondents in all workforce groups who were receptive to both online and face-to-face modes of delivery. Of those who indicated a preference, there was a significant preference (Kruskal-Wallis p < 0.001) for online learning (n = 861) over face-to-face learning (n = 653). The remaining respondents (n = 1025) indicated that they were receptive to both types of learning. There were no significant differences between workforce groups in preferred training delivery methods.

Several respondents provided comments in the questionnaire about barriers to accessing continuing professional development (CPD) opportunities. The most common theme was a lack of protected time to participate in CPD.

*“I am using my annual leave to do my further training in genomics as the (hospital) does not provide any training or allow study leave for this reason”* (Junior Doctor, Foundation year training)

*“If spaces are made available … there is no capacity within the (hospital) to allow time to train—understaffing, under resourced, plus not enough study days”* (Healthcare Scientist, Genomics).

In some cases, this appeared to pertain to accessing protected time to access online courses.

*“Can't get study leave for online learning”* (Medical Consultant, Pediatrics)

A number of respondents also raised the issue of a lack of funding to pay for the education or training session.

*“I like the idea of learning more, but I don't have the time, energy or funds”* (Clinical Researcher)

Five surveys (n = 1,786 respondents) also provided a list of reasons that may motivate individuals to undertake training: continuing professional development (84.6%, n = 1,511) and direct impact on patient care (71.8%, n = 1,283) were the reasons most often cited. There were no significant differences between workforce groups.

## Discussion

This paper reports the perceived education and training needs in genomics of England's NHS staff, the largest assessment of this workforce to date. The aim of this work was to collect data from NHS staff that could be used to direct the work of local education and training initiatives and that of the GEP. As with all surveys there is the potential for response bias. Due to the nature of how these surveys were deployed there will be a level of response bias, with people with a vested interest in the subject more likely to respond (Duda and Nobile, 2010). However, views have been collected from a diverse group of staff, not all of whom were familiar with genomics or used genomics within their current role.

Not all respondents identified a need for genomics education and training, but the proportion who expressed a need differed depending on how the question was asked. When asked if they have sufficient genomics knowledge and skills to perform their current role, the proportion of respondents who responded “no,” therefore indicating a need for education and training, was much lower than when a general question was asked about engaging in genomics education and training activities. These responses suggest that there is an appetite for genomics education and training initiatives within the NHS, even if this knowledge and/or the skills are not yet required by an individual to undertake their job role. In most cases there was no significant difference observed between the different workforce groups and their perceived education and training needs, and perceived need was not significantly influenced by previous education and training.

The identification of the four different types of genomic education and training needs provides a framework in which to segment the NHS workforce on their learning requirements rather than their workforce group. Each segment of the workforce has differing requirements.

- *Those who understand their role in genomics and feel they are adequately equipped now*. These individuals are likely to need updates as the science evolves and how genomics is implemented within the NHS changes.
- *Those who understand their role in genomics and have a specific learning need*. These individuals will need access to resources to help them close their knowledge or skill gap.
- *Those who do not fully understand how genomics relates to their role*. These individuals identified a need for more general information about genomics so they can identify how this technology impacts on their role, and the patients that they care for.
- *Those who do not see genomics as being relevant to their role, and so do not think there is a need to learn about it*. While some NHS staff in this group may not require an understanding of genomics to perform their role, others will. This second group is likely to be the most challenging group to reach as they will need persuading of the relevance of genomics to their work before they will engage in any relevant learning.

### Informing Genomic Education and Training Resource Development

For those NHS staff that need to understand genomics and apply this to their practice, our findings suggest there are two levels of education and training resources required. The first is general information targeted to professional groups and the second is cross-professional resources on specific areas or activities that form part of the clinical pathway. However, the results from these surveys also emphasize the need for ongoing awareness raising about genomics in general, as there are still healthcare professionals, as well non-clinical NHS staff, who do not know what genomics is, let alone how it can be applied to healthcare.

**Figure 1:**
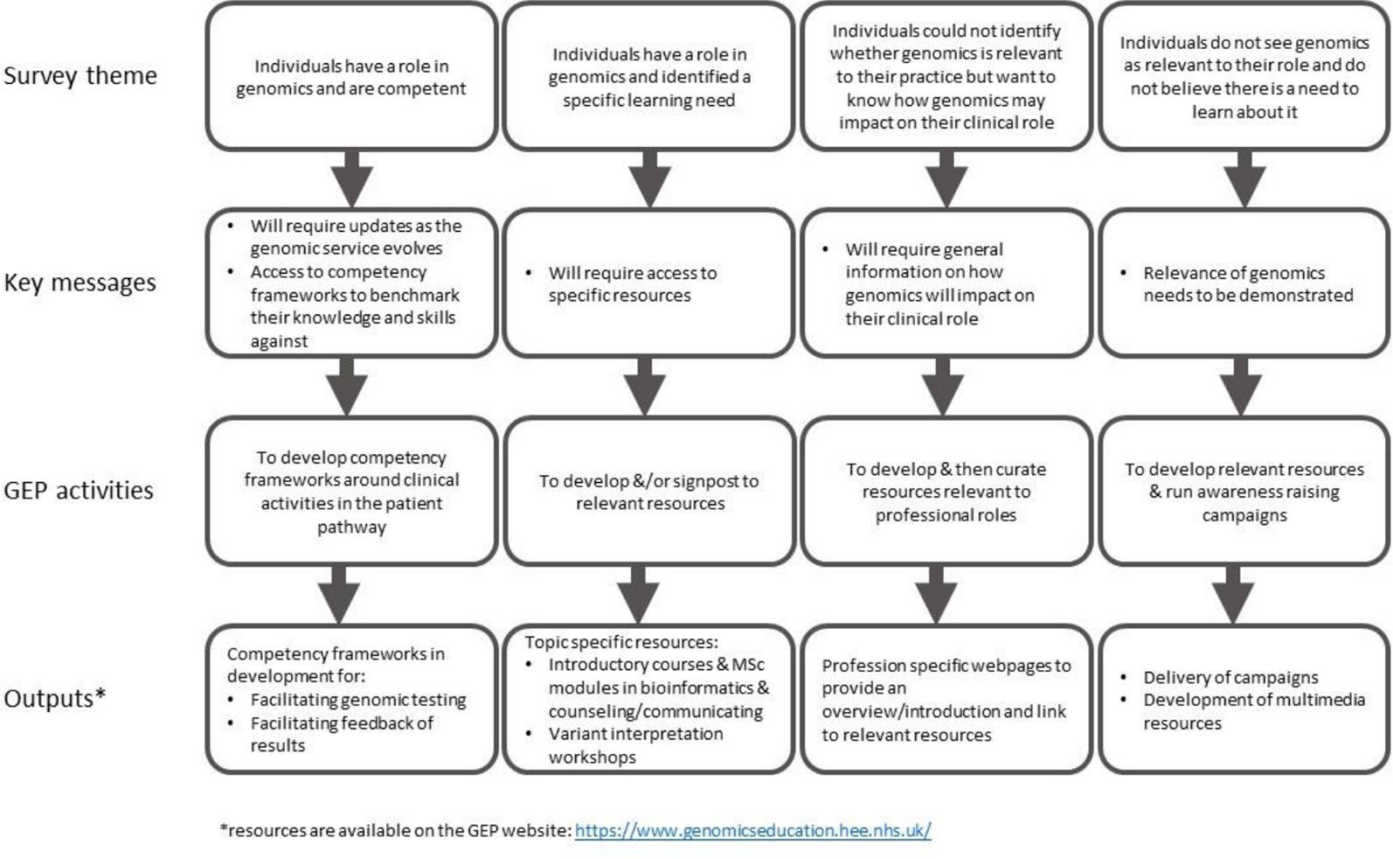
Process taken from survey results to resource development.

These findings have influenced the development of GEP resources, addressing both levels of education and training requirements, as well as general awareness, with innovative ways to engage and inform our audiences, ranging from videos and animations to formal qualifications (for example, Master's level). **Figure 1** demonstrates how key messages from each of the themes have guided GEP activities and outputs. Resources targeting specific professional groups highlighting where and how genomics is relevant in these clinical areas have been produced (www.genomicseducation.hee.nhs.uk/genomics-in-healthcare/). Cross-professional education and training resources corresponding to clinical activities across the patient pathways in the new Genomic Medicine Service are also in development. In addition to delivering education and training resources, the GEP has initiated the development of cross-professional competencies. Work has commenced on defining these competencies for the clinical activities of the consent conversation and feedback of genomic test results. These competencies can direct future work of the GEP, by prioritizing resource development, and they can support individual NHS staff by providing a framework that they can use to identify learning or training gaps (Hepp et al., 2015).

The importance of providing the NHS workforce with these two levels of education and training resources has also been recognized at a policy level. The Interim NHS People Plan, which sets out how people working in the NHS will deliver the ambitious 10-year vision for healthcare in England, signals the need for a NHS workforce that has education and training “tailored to the needs of the individual” and with a balance of general knowledge and specialist skills depending on the clinical role (NHS England, 2019a).

### Supporting NHS Staff to Engage in Education and Training

NHS staff overall showed a significant preference for online delivery; however, it is important to note that many respondents still preferred face-to-face education and training. It is unclear from our results if individual's preference is due to personal learning styles or more pragmatic reasons such as their ability to access to learning. It is recognized that some training, such as learning practical skills, including laboratory science, may be best delivered face-to-face (Jaggars, 2014). However, there are times when online learning is equally or more effective than face-to-face delivery and often has the added advantage of being flexible, allowing learners to access learning opportunities at a time and place that suits them (Maloney et al., 2015; Brady et al., 2018).

Providing different modes of delivery allows individuals to choose the method that best serves them, either in terms of learning style or time and convenience, but this may not always be possible. In the case of genomics education within the NHS, the scale and pace at which education and training needs to occur often makes online learning the most practical choice for those developing resources. While there is recognition at a national level that access to continuing professional development is a priority for the NHS (NHS England, 2019a), our findings suggest people are becoming less willing to do CPD in their own time. Concessions will therefore be needed to be made to ensure the same consideration for protected learning time is given for those wanting to participate in online learning rather than face-to-face sessions.

### Understanding Motivations to Engage in Learning and Applying This to Resource Design

Understanding training motivations can help ensure education and training courses and resources are appropriately marketed to the audience. However, an individual's education or training motivation can also influence the depth to which they will learn. Training motivators can be considered intrinsic or extrinsic, but these are not mutually exclusive. Individuals primarily motivated by intrinsic factors are likely to be deep learners, while individuals motivated by extrinsic factors are typically surface learners (Baeten et al., 2010). As an educator, understanding target audience's motivations can help tailor content to maximize learning. For example, individuals who are undertaking training purely to meet CPD requirements are likely to be, at least initially, less engaged surface learners, learning what they need to pass, compared to individuals who are undertaking training because they are motivated by intrinsic factors such as “direct impact on patient care”.

While meeting CPD requirements was the main motivator of our respondents, there were many NHS staff who identified “direct impact on patient care,” an intrinsic factor, as a primary motivation to engage in learning. This suggests this proportion of the workforce will be deep learners if they can see how learning will benefit their patients. Understanding these two factors has influenced the way in which the GEP develops its resources. Where relevant, education and training activities are accredited with relevant bodies as recognized CPD activities. In addition, the GEP ensures that the link between the learning activity and patient care is a central component in resource development.

## Conclusions

The findings from these surveys have provided an evidence base that informs the ongoing strategy for the GEP. This study demonstrates how a questionnaire-based needs assessment can provide information to direct the development of relevant resources to meet the education and training needs of a diverse health professional workforce.

The development of evidence-based competency frameworks and educational resources by the GEP to support all NHS staff who will use genomics as part of their role in the patient pathway will result in a workforce better placed to take advantage of advances in genomic medicine.

## Data Availability Statement

The datasets generated for this study are available on request to the corresponding author.

## Ethics Statement

Ethical review and approval was not required for the study on human participants in accordance with the local legislation and institutional requirements. The patients/participants provided their written informed consent to participate in this study.

## Author Contributions

MB conceived the idea for publication. MB and SS had intellectual input into the study design. MB and SS contributed to data analysis. MB, SS, and AS provided intellectual input into preparation of the manuscript. All authors approved the final version and agree to be accountable for all aspects of the work.

## Funding

This work was supported by Health Education England's Genomics Education Programme.

## Conflict of Interest

The authors declare that the research was conducted in the absence of any commercial or financial relationships that could be construed as a potential conflict of interest.

## Supplementary Material

Supplementary Figure 1 Map showing approximate geographical regions covered by each GMC.
