# Supplementary material for: Using the Findings of a National Survey to Inform the Work of England’s Genomics Education Programme
Source: Front Genet. 2019 Dec 17;10:1265. doi: 10.3389/fgene.2019.01265 (PMC6927929; doi:10.3389/fgene.2019.01265)
Supplement: Supplementary file 1 [file DataSheet_1.docx]

**Quotes from each of the four themes relating to NHS staff’s education and training needs.**

1. *Individuals have a role in genomics and are competent*. These individuals felt they had enough knowledge and the right skills to perform their current role, however respondents were cognisant that genomic knowledge constantly evolves:

- ‘There’s always so much to learn’ (Nurse, Pediatrics)
- ‘Service is expanding rapidly therefore important to keep up-to-date with current tests and techniques.’ (Clinical Scientist, Genetics)
- ‘I would always welcome more skills in this area. At present I feel I have enough skills to perform effectively, but there is always room for improvement’ (Medical, Pediatrics)
- ‘But I may be unconsciously incompetent’ (Medic, Immunology)
- ‘Although I say yes (I have enough knowledge), I am worried that I may be missing some information. I don’t know what I need to know especially for the future. What I know now is enough for now.’ (Medic, Oncologist)

1. *Individuals have a role in genomics and identified a specific learning need*. All of these individuals had previously indicated that they were involved in genomics, and the comments below are exemplar quotes providing examples of specific learning needs:

- ‘Genetic reports are largely incomprehensible to non-geneticists. I would like a better understanding of them’ (Medic, Pediatrician)
- ‘I need better bioinformatics skills’ (Medic, Clinical Genetics)
- ‘I could expand my role if I had further bioinformatics training but contribute within my competencies’ (Medic, Immunology)
- ‘I would benefit from applied bioinformatics training’ (Healthcare Scientist, Pathologist)
- ‘Would like to be better informed in new methods of genetic sequencing (Medic, Dermatology)
- ‘I am fully versed in molecular tests for mutational analysis, but I do not feel able to counsel patients (Medic, Respiratory medicine)
- ‘How will it affect the procuring, storage and distribution of medicines. Will pharmacists' counselling of patients be altered?’ (Pharmacist)
- ‘Don’t understand enough about genetic counseling’ (Medic, Primary Care)
- ‘Advice on consenting issues and how results will be fed back to patients’ (Nurse, Pediatrics)
- ‘Whilst I understand the implications of specific genetic conditions I see every day in my caseload would like more genetic counselling training’ (Nurse, Neurology)

1. *Individuals could not identify whether genomics is relevant to their practice but want to know how genomics may impact on their clinical role.* Some of these respondents were aware that genomics would be relevant to their professional group, whereas others were not sure. However, both groups still wanted to find out more about the application of genomics to healthcare. In general, these respondents requested introductory level resources, primarily related to their professional group:

- ‘Genomics for nurses’ (Nurse, specialism not specified)
- ‘Genomics for Rheumatology (Pharmacist, Rheumatology)
- ‘Application with respect to radiology.’ (Medic, Radiologist)
- ‘Genomics as it relates to Stroke’ (Medic, Stoke specialist)
- ‘Genetics could influence the way we treat patients’ pain. Although I have no training in genomics, I’m open to learning how this can influence the future of our patients (Nurse, Acute pain)
- ‘I would be keen to relate it to my specialty if that is possible.’ (Pharmacy, Psychiatry)

1. *Individuals do not see genomics as relevant to their role and do not believe there is a need to learn about it*. These NHS staff were not interested in knowing more about genomics, as they could not see how it would change their every-day practice:

- ‘Do I need to know more? I can do my job without having any knowledge in genomics’ (intensive care nurse)
- ‘I don’t think it has anything to do with my job’ (plastics and orthopedics)
- ‘I do not have any dealings in the role that I do within the Finance Team’ (Admin and Clerical)
- ‘Knowledge not required for my job’ (Midwifery)
