# Supplementary figures and images for: Using the Findings of a National Survey to Inform the Work of England’s Genomics Education Programme
Source: Front Genet. 2019 Dec 17;10:1265. doi: 10.3389/fgene.2019.01265 (PMC6927929; doi:10.3389/fgene.2019.01265)

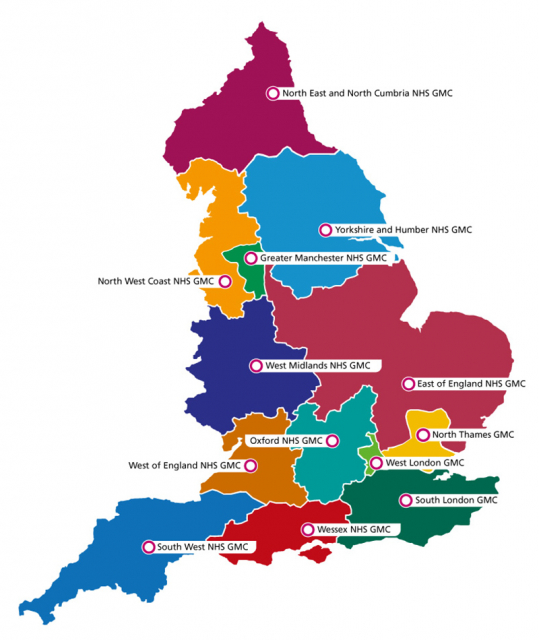

Supplement: Supplementary Figure 1 — Map showing approximate geographical regions covered by each GMC. [file Image_1.jpg]
